# Absence of Sigma 1 Receptor Accelerates Photoreceptor Cell Death in a Murine Model of Retinitis Pigmentosa

**DOI:** 10.1167/iovs.17-21947

**Published:** 2017-09

**Authors:** Jing Wang, Alan Saul, Xuezhi Cui, Penny Roon, Sylvia B. Smith

**Affiliations:** 1 Department of Cellular Biology and Anatomy, The Medical College of Georgia at Augusta University, Augusta, Georgia, United States; 2 The James and Jean Culver Vision Discovery Institute, Augusta University, Augusta, Georgia, United States; 3 Department of Ophthalmology, Augusta University, Augusta, Georgia, United States

**Keywords:** rd10 mouse, retinal neuroprotection, ERG, retinal degeneration, rods, cones

## Abstract

**Purpose:**

Sigma 1 Receptor (Sig1R) is a novel therapeutic target in neurodegenerative diseases, including retinal disease. *Sig1R^−/−^* mice have late-onset retinal degeneration with ganglion cell loss that worsens under stress. Whether Sig1R plays a role in maintaining other retinal neurons is unknown, but was investigated here using *rd10* mice, a model of severe photoreceptor degeneration.

**Methods:**

Wild-type, *rd10*, and *rd10*/*Sig1R^−/−^* mice were subjected to ERG and spectral-domain optical coherence tomography (SD-OCT) to assess visual function/structure in situ. Retinas imaged microscopically were subjected to morphometric analysis, immunodetection of cones, and analysis of gliosis. Oxidative and endoplasmic reticulum (ER) stress was evaluated at mRNA/protein levels.

**Results:**

Photopic ERG responses were reduced significantly in *rd10*/*Sig1R^−/−^* versus *rd10* mice at P28 (31 ± 6 vs. 56 ± 7 μV), indicating accelerated cone loss when Sig1R was absent. At P28, SD-OCT revealed reduced retinal thickness in *rd10*/*Sig1R^−/−^* mice (60% of WT) versus *rd10* (80% of WT). Morphometric analysis disclosed profound photoreceptor nuclei loss in *rd10*/*Sig1R^−/−^* versus *rd10* mice. *rd10*/*Sig1R^−/−^* mice had 35% and 60% fewer photoreceptors, respectively, at P28 and P35, than *rd10*. Peanut agglutinin cone labeling decreased significantly; gliosis increased significantly in *rd10*/*Sig1R^−/−^* versus *rd10* mice. At P21, NRF2 levels increased in *rd10*/*Sig1R^−/−^* mice versus *rd10* and downstream antioxidants increased indicating oxidative stress. At P28, ER stress genes/proteins, especially XBP1, a potent transcriptional activator of the unfolded protein response and CHOP, a proapoptotic transcription factor, increased significantly in *rd10*/*Sig1R^−/−^* mice versus *rd10*.

**Conclusions:**

Photoreceptor cell degeneration accelerates and cone function diminishes much earlier in *rd10*/*Sig1R^−/−^* than *rd10* mice emphasizing the importance of Sig1R as a modulator of retinal cell survival.

The major cause of untreatable blindness worldwide is retinal degenerative disease, most often due to death of photoreceptor or ganglion cells (RGCs).^1^ Retinitis pigmentosa (RP), a photoreceptor cell (PRC) degenerative disease, affects 1:3000 to 5000 people.^2^ Initially, rod PRCs are lost compromising vision in dim light (nyctalopia); slow, insidious loss of the visual field eventually leads to “tunnel vision.” Assessment of visual function by ERG reveals an extinguished scotopic (dark-adapted) response reflecting rod dysfunction. Subsequent cone loss, reflected as a decline of visual acuity, decreased vision in bright light, and diminished photopic ERG, is the most debilitating consequence of RP.^2–4^ If strategies can be developed to preserve cone function, even when rods are lost, the therapeutic impact on this devastating disease would be enormous.

RP is caused by more than 3000 mutations in more than 50 different genes.^5^ Given its heterogeneity, developing treatments for RP aimed at common disease mechanisms may be more successful than targeting specific genetic defects. Useful in this endeavor is the availability of models of RP, including the *Pde6b^rd10^/J (rd10)* mouse. *Rd10* mice carry a spontaneous missense point mutation in exon 13 of the beta-subunit of rod cyclic guanosine monophosphate (cGMP) phosphodiesterase (β-PDE) gene.^6–8^ Mice lose rod PRCs beginning at postnatal day 18 (P18), the rod cell loss peaks at P25. Cone cell death occurs in this model. By P35 most cones are lost and the cone function is minimal.^8^

We recently investigated the consequences of activating the sigma 1 receptor (Sig1R) in attenuating cone cell loss in the *rd10* mouse.^9^ Sig1R is a 25.3-kDa transmembrane receptor protein. Unlike many transmembrane receptors belonging to large, extensively studied families (e.g., G-protein-coupled receptors, ligand-gated ion channels), Sig1R is an evolutionary isolate with no discernible similarity to other proteins. Its recently published crystal structure in humans reveals a trimeric architecture with a single transmembrane domain in each protomer.^10^ Sig1R, a putative molecular chaperone,^11^ is an enigmatic protein whose precise physiological function is unknown. It is emerging as a unique pluripotent modulator of cell survival that interacts with many structurally diverse proteins.^12–14^ Mutations of *SIG1R* are implicated in human neurodegenerative diseases, including amyotrophic lateral sclerosis, Alzheimer's disease, and Parkinson's disease,^15–20^ whereas activating Sig1R has conferred neuroprotection in animal models of these diseases.^21–23^

In addition to expression in the central nervous system, *Sig1R* is expressed in multiple retinal cell types, including PRCs, RGCs, and Müller and pigment epithelial cells.^24–28^ Several laboratories reported that Sig1R activation attenuates RGC death,^29–34^ mitigates retinal glial cell reactivity,^35–37^ and diminishes light-induced PRC loss.^38^ We evaluated effects of activation of Sig1R in *rd10* mice following administration of (+)-pentazocine ([+]-PTZ), a high-affinity Sig1R ligand.^9^ Photopic (cone) ERGs obtained at P35 showed that b-wave amplitudes were significantly improved compared with nontreated animals. We then subjected mice to an electrophysiological test termed the pseudorandom luminance noise test, which provides a light stimulus that is more similar to natural vision.^39^ Using this test, we observed responses in (+)-PTZ–treated *rd10* mice that were similar to wild type (WT) suggesting significant rescue of cones in mutant animals.^9^ When we examined retinal histologic sections from these mice using immunohistochemical methods, we determined that the functional improvement in cone function was associated with attenuated cone cell death as noted by increased labeling using the cone-specific markers cone arrestin and peanut agglutinin (PNA). Protective effects of (+)-PTZ were attributable to Sig1R activation because cone function and structure were not preserved in (+)-PTZ–treated *rd10* mice that lacked Sig1R (*rd10*/*Sig1R^−/−^* mice).^9^ Whether the rate or severity of PRC degeneration characteristic of *rd10* mice was altered in *rd10*/*Sig1R^−/−^* mice was not investigated.

Assessment of consequences of Sig1R deletion on mouse retina reveal normal development and no overt retinal phenotype or dysfunction through 6 months.^40^ By 1 year, however, *Sig1R^−/−^* mice develop a late-onset loss of RGC function,^40^ which is accelerated when the mice are made diabetic^41,42^ or subjected to optic nerve crush.^43^ Interestingly, although RGC death worsens in *Sig1R^−/−^* mice, the remainder of the retina is not affected. There is no alteration in outer nuclear layer (ONL) thickness nor apparent loss of PRCs. In the current study, we were interested in determining the consequences on retina function and morphology when mice lacking *Sig1R* were bred to homozygosity with *rd10* mice. Given that Sig1R may be a modulator of cell survival,^12–14^ we investigated rate/severity of PRC loss in *rd10* mice lacking *Sig1R* (*rd10*/*Sig1R^−/−^*) beginning at P15, age that precedes PRC loss in *rd10* mice.

## Methods

### Animals

A total of 272 mice were used in the study (Supplementary Table S1). All animals were maintained according to guidelines of the Institutional Animal Care and Use Committee at Augusta University and the ARVO Statement for Use of Animals in Ophthalmic and Vision Research. Animals were subjected to standard light cycles (12 hours light:12 hours dark) and the light level measured from the bottom of cages was approximately 10 to 15 lux. Breeding pairs of homozygous *rd10* mice (B6.CXBI-*Pde6βrd10/J*) were shipped from The Jackson Laboratory (Bar Harbor, ME, USA) to the animal facility of Augusta University. To confirm the genotype of *rd10* mice, PCR was performed using the following primers to amplify genomic DNA: *Pde6b* forward 5′-CTTTCTATTCTCTGTCAGCAAAGC-3′ and reverse 5′-CATGAGTAGGGTAAACATGGTCTG-3′. Amplification was followed by *CfoI* enzyme digestion per the method of Chang et al.^7^ The generation of *Sig1R*^−/−^mice has been described^44^ and retinal phenotype of homozygous (*Sig1R*^−/−^) mice has been documented comprehensively.^40^ *Pde6βrd10*/J mice were bred with *Sig1R*^−/−^ mice to produce *Pde6βrd10*^+/−^/*Sig1R*^+/−^ mice, which were crossed to generate *Pde6βrd10*/*Sig1R*^−/−^ mice (hereafter referred to as *rd10/Sig1R^−/−^* mice). Absence of *Pde6β* and *Sig1R* was confirmed by genotyping.^9^ The *rd10* and *Sig1R^−/−^* mice are on the C57BL/6 background, thus C57BL/6J mice (Jackson Laboratory) were used as WT controls. Mice were screened also for the Crb1^rd8/rd8^ mutation and were negative. The Crb1^rd8/rd8^ mutation, which causes focal disruption of the retina, has been reported in some mouse strain stocks; it is essential to exclude the Crb1^rd8/rd8^ mutation before studying new retinal models.^45^

### Electroretinogram

ERGs were obtained from dark-adapted mice anesthetized with isoflurane. Silver-coated nylon fibers joined to flexible wires were carefully placed on the cornea, and a drop of hypromellose coated the eye and provided improved electrical contact. Optic fibers of 1 mm diameter were positioned just in front of the pupils. A 5500° white light-emitting diode provided highly controllable illumination that was led to the eyes through the optic fibers. Experiments consisted of a series of tests with 5-ms flashes of increasing luminance, followed by photopic testing with 5-ms flashes above a pedestal, as well as other photopic stimuli that included a “natural” noise stimulus. The natural noise was a slowly varying luminance time series with amplitude inversely proportional to temporal frequency, termed the pseudorandom luminance noise test.^39^ Data were averaged across the two eyes for each animal, then averages were computed for each group across the animals. Kernels were computed from the responses to the natural noise stimulus by correlating the responses with the stimuli.

### Spectral-Domain Optical Coherence Tomography (SD-OCT)

The integrity of the retina was assessed in vivo using SD-OCT. Mice were anesthetized using a rodent anesthesia cocktail containing ketamine 100 mg/mL, xylazine 12 mg/mL (Sigma-Aldrich Corp., St. Louis, MO, USA). Pupils were dilated with 1% tropicamide (Bausch & Lomb, Tampa, FL, USA) followed by application of GenTeal Lubricant Eye Gel (Alcon, Ft. Worth, TX, USA). Systane lubricant eye drops (Alcon) were applied throughout the procedure to keep the cornea moist. SD-OCT images were obtained using the Bioptigen Spectral-Domain Ophthalmic Imaging System (Envisu R2200; Bioptigen, Morrisville, NC, USA). Imaging included averaged single B scan and volume intensity scans (VIP) with images centered on the optic nerve head. Postimaging analysis included auto segmentation report analysis and manual assessment of all retinal layers using InVivoVue Diver 2.4 software (Bioptigen). The OCT data from *rd10* mice younger than P21 could be processed using autosegmentation report analysis; however, OCT data from *rd10* mice older than P21 required manual segmentation analysis. We measured total retinal thickness (TRT), thickness of nerve fiber layer thickness, inner plexiform layer (IPL), inner nuclear layer (INL), outer plexiform layer (OPL), ONL, inner/outer segment thickness, and RPE thickness. Each layer thickness was plotted separately, and the data for a given retinal layer in each group were averaged.

### Histologic Processing of Tissue, Microscopic Evaluation, and Morphometric Analysis

Eyes, enucleated from euthanized mice, were prepared for cryosectioning or embedding in JB-4 methacrylate (Electron Microscopy Sciences, Hatfield, PA, USA). For cryosections, eyes were flash frozen in liquid nitrogen and embedded in optimal cutting temperature compound (Tissue-Tek, Sakura Finetek, Torrance, CA, USA); 10-μm-thick cryosections were fixed 10 minutes in 4% paraformaldehyde (4% PFA), and blocked with 10% goat serum in 0.1% Triton X-100/PBS for 1 hour at room temperature. For plastic embedding, eyes were immersion-fixed in 2% paraformaldehyde/2% glutaraldehyde in 0.1M cacodylate buffer and processed for JB-4 embedding. Sections were stained with hematoxylin-eosin (H&E), retinal images captured using an Axioplan-2 microscope equipped with a high-resolution camera, and processed using Zeiss Axiovision software (version 4.7; Carl Zeiss, Oberkochen, Germany). Retina sections were scanned first for gross disruption followed by comprehensive morphometric evaluation. Retinal thickness; thickness of INL, ONL, IPL, OPL, and inner/outer segments; and number of RGCs/100 μm retinal length were determined in six images captured temporal/nasal to optic nerve, yielding data for central, midperipheral, and peripheral retina.

### Immunofluorescence Studies in Retinal Cryosections and Flatmounted Retinas

Retinal cryosections were incubated with primary antibodies (anti-cone, anti-glial fibrillary acidic protein [GFAP]) or FITC-conjugated PNA followed by incubation with secondary antibodies (Supplementary Table S2). Oxidative stress was detected in retinal cryosections using chloromethyl derivative of 2′,7′-dichlorodihydrofluorescein diacetate (CM-H_2_DCFDA) (C6827; Molecular Probes/Thermo Scientific, Eugene, OR, USA) following the manufacturer's protocol. For flatmounted retinal preparations, eyes were enucleated, fixed in 4% PFA, and washed with PBS. The neural retina was dissected, incubated with 0.3% Triton X-100 in PBS, and blocked with 10% goat serum. Samples were incubated with FITC-PNA or with anti-Iba1 at 4°C overnight followed by incubation with secondary antibodies. Retinas were then cut partially with sharp scissors to create petals allowing the retina to be flattened and placed on a microscope slide. Retinas were examined using a Zeiss LSM 780 upright laser-scanning confocal microscope equipped with ZEN lite software. Retinal areas (measuring 0.12 mm^2^) located 0.5 mm superior, inferior, temporal, and nasal to the center of the optic nerve were photographed, and positive cells quantified using Image J 1.48v software (http://imagej.nih.gov/ij/; provided in the public domain by the National Institutes of Health, Bethesda, MD, USA).

### Analysis of ER Stress and Oxidative Stress–Related Genes and Proteins

Expression levels of mRNA transcripts specific for several key genes involved in oxidative stress (*Nrf2, Keap1, Sod1, Catalase, Nqo1, Gpx1, Hmox-1*, and *Gsst3*) and endoplasmic reticulum (ER) stress (*Ire1α*, *Xbp1*, *Atf4*, *Chop*, *Bip*, *Perk*, *Ip3r3*, and *Atf6*) were examined in neural retina of *rd10/Sig1R^−/−^*, *rd10* mice compared with age-matched WT mice. Total RNA was purified from isolated neural retinas using Trizol (Invitrogen, Carlsbad, CA, USA) according to the manufacturer's protocol and quantified. RNA (2 μg) was reverse-transcribed using the iScript Synthesis kit (BioRad Laboratories, Hercules, CA, USA). cDNAs were amplified for 40 cycles by using SsoAdvanced SYBR Green Supermix (BioRad Laboratories) and gene-specific primers (Supplementary Table S3) in a CFX96 Touch Real-Time PCR Detection System (BioRad Laboratories). Expression levels were calculated by comparison of Ct values (delta-delta Ct).^36^ Protein was extracted from neural retina and subjected to SDS-PAGE; nitrocellulose membranes, to which the proteins had been transferred, were incubated with primary antibodies (Supplementary Table S2). Immunoblotting was performed to assess oxidative stress–related proteins (NRF2, KEAP1, SOD1, Catalase, NQO1, HMOX1) and ER stress–related proteins (IRE1α, XBP1, ATF4, CHOP, BIP/GRP78, PERK, IP3R3). Proteins were visualized using the SuperSignal West Pico Chemiluminescent Substrate detection system (Pierce Biotechnology, Waltham, MA, USA). Band densities were quantified using Image J 1.48v software.

### Statistical Analysis

Statistical analysis was conducted using the GraphPad Prism analytical program (LaJolla, CA, USA), or for the ERG results, Igor Pro (Lake Oswego, OR, USA). Significance was established as *P* < 0.05. Data were analyzed by 1-way ANOVA for studies comparing one parameter among the three mouse groups or 2-way ANOVA (factors: mouse group and retinal region measured; mouse group and gene expression). We followed the recommendations for appropriate post hoc testing, which included Holm-Bonferroni, Tukey's, and Newman-Keul's Multiple Comparison Tests. Graphic representation of data significance was depicted as *P < 0.05; **P < 0.01; ***P < 0.001.

## Results

### Sig1R Deletion Compromises Photoreceptor Function in *rd10* Mice

**Figure 1:**
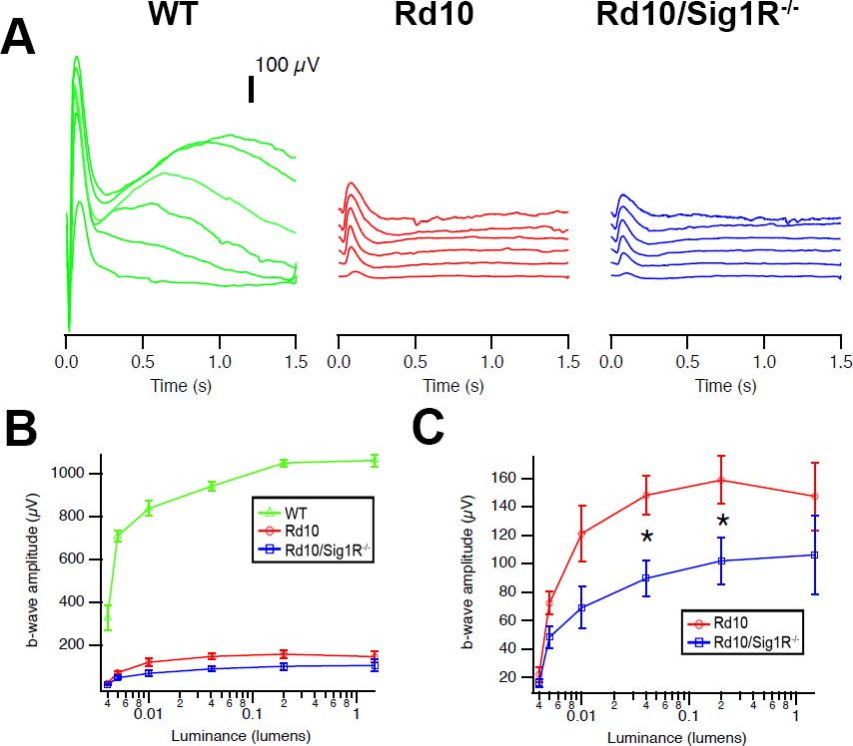
Scotopic ERG. (A) Scotopic ERG traces. Averaged scotopic ERG responses to 5-ms flashes at a series of intensities in WT, rd10, and rd10/Sig1R^−/−^ mice at P28; intensities are units of lumens. (B) Mean b-wave amplitudes. Data are the mean ± SEM of assessments of six to nine mice. (C) Enlargement of b-wave amplitude in rd10 and rd10/Sig1R^−/−^. *Significant difference between rd10 and rd10/Sig1R^−/−^ mice (P < 0.05).

**Figure 2:**
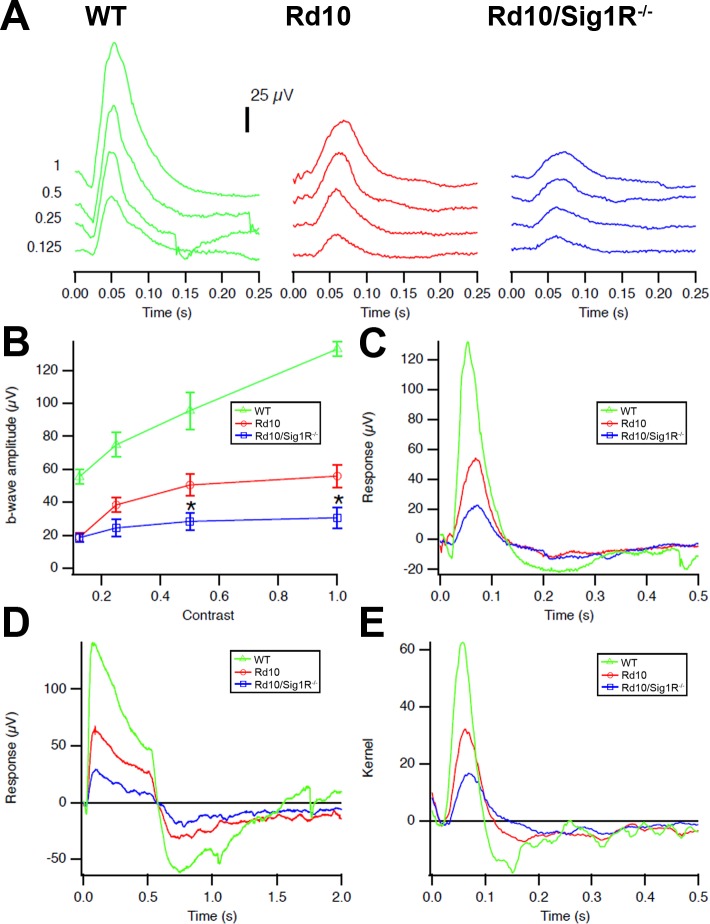
Photopic ERG and natural luminance noise test. (A) Photopic ERG traces. Averaged photopic responses to 5-ms flashes at a series of contrasts are provided for WT, rd10, and rd10/Sig1R^−/−^ mice at P28. (B) Mean b-wave amplitudes of averaged photopic responses to 5-ms flashes above a fixed pedestal luminance of 0.105 lumens (four contrasts of the flash; contrast = [flash-pedestal]/pedestal luminance). Data are the mean ± SEM of four assays using eyes from six to nine mice. *Significantly different from the WT and rd10 groups, *P < 0.05. (C) Averaged responses to photopic flash of contrast =1 (replotted after superimposition). (D) Averaged responses to 0.5-second-long luminance steps are shown. The photopic negative response occurs after stimulus offset at 0.5 second, and originates in part from RGCs. (E) Averaged kernels derived from responses to natural noise stimuli. Green, WT mice; red, rd10 mice; blue, rd10/Sig1R^−/−^ mice.

Scotopic ERGs performed on WT mice at P28 showed predictably robust responses (Fig. 1A) with b-wave amplitudes of 1000 μV at higher luminance intensities; *rd10* mice had reduced responses averaging less than 200μV (Fig. 1B). There was significantly less rod responsiveness (<100 μV) in *rd10/Sig1R^−/−^* mice compared with *rd10* mice at P28 (Fig. 1C). Cone function of WT mice was robust at P28 (Fig. 2A), with b-wave amplitudes greater than 100 μV as light intensity increased. *rd10* mice showed detectable cone activity (approximately 40 μV) (Fig. 2B), whereas cone responses in P28 *rd10/Sig1R^−/−^* mice were reduced significantly compared with *rd10* mice (approximately 20 μV) (Fig. 2C). P28 cone responses were evaluated using the pseudorandom luminance noise test (Figs. 2D, 2E). *rd10* mice responded with approximately half-WT levels, whereas *rd10/Sig1R^−/−^* mouse responses were reduced dramatically compared with *rd10* mice (Figs. 2D, 2E). These functional studies demonstrate accelerated PRC dysfunction, particularly cones, in *rd10* mice lacking Sig1R.

### Sig1R Deletion Accelerates Retinal Cell Loss in *rd10* Mice

**Figure 3:**
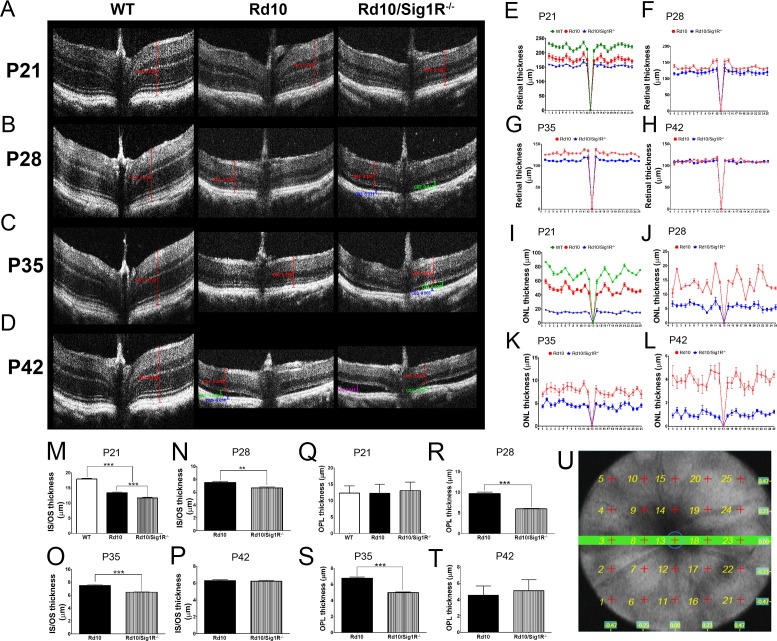
SD-OCT assessment. (A–D) Representative SD-OCT data obtained from WT mice, rd10 mice, and rd10/Sig1R^−/−^ mice at P21 (A), P28 (B), P35 (C), and P42 (D). (E–T) Data for segmentation analysis for TRT at P21, P28, P35, and P42 (E–H), for ONL thickness at P21 to P42 (I–L), for IS/OS thickness at P21 to P42 (M–P), and for OPL thickness at P21 to P42 (Q–T). (U) The template (5 × 5 grid) for the OCT measurements. It represents the 25 points within the retina where retinal thickness is measured. Data are the mean ± SEM of analyses in 4 to 10 mice per group at each age. **P < 0.01, ***P < 0.001. Note: For the OCT analyses, the x-axis represents the 25 points shown in (U), with point #13 representing the optic nerve, where the thickness is always zero.

Consequences of Sig1R deletion on retinal structure in *rd10* mice in vivo, monitored by SD-OCT, showed that over the period of study (P21–P42), TRT of WT mice averaged approximately 250 μm (Figs. 3A–D). In *rd10* mice (P21), TRT was 80% that of WT (approximately 200 μm), whereas in *rd10/Sig1R^−/−^* mice, it was 60% that of WT (approximately 150 μM). TRT in *rd10/Sig1R^−/−^* mice was significantly less than *rd10* mice through P35 (Figs. 3E–G), suggesting accelerated retinal degeneration in mice lacking Sig1R. Only at P42 was the TRT equivalent between *rd10* and *rd10/Sig1R^−/−^* mice (Fig. 3H). The decrease in *rd10* TRT by P42 is consistent with earlier reports.^46^

ONL thickness (ONL-T) measured by SD-OCT in WT mice ranged from approximately 65 to 85 μm over the period analyzed. *rd10* mice had significantly reduced ONL-T of approximately 40 to 60 μm at P21; ONL-T diminished further in *rd10/Sig1R^−/−^* mice to only approximately 20 μm (Fig. 3I). Over the time course studied, P28, P35, P42, the *rd10* ONL-T decreased from approximately 12 to approximately 7 to approximately 4 μm, respectively (Figs. 3J–L). Decreased ONL-T accelerated dramatically in *rd10/Sig1R^−/−^* mice (approximately 5 μm at P28 and P35; only approximately 2 μm by P42 [Figs. 3J–L]). The inner/outer segments typically measure approximately 15 μm in WT mice and approximately 12 μm in *rd10* (Fig. 3M). The thickness was reduced significantly in *rd10/Sig1R^−/−^* mice versus *rd10* at P21, P28, and P35 (Figs. 3N–P). It has been reported by others that as the PRC loss becomes severe in *rd10* mice (i.e., after P35 when there is less than one row of nuclei), the remaining retina may separate from the underlying RPE.^7–9^ An OCT analysis of *rd10* mice reported by Hasegawa and colleagues^46^ noted approximately 30% of mice demonstrated this phenomenon. We observed a similar trend in *rd10* mice by P35, whereas we observed this earlier (beginning at P28) in *rd10/Sig1R^−/−^*mice. Note that the data shown in Figures 3E–T represent 25 individual points within the retina where measurements are made. The software provided by the manufacturer reports data as a 5 × 5 grid. The 25 separate points (with the optic nerve head in the center #13) are depicted in Figure 3U.

The OPL is composed of synapses between PRCs and INL cells. It is approximately 10-μm thick in WT mice (Fig. 3Q). *rd10* mice maintain an OPL similar to WT through P28, with gradual reduction by P35 (Figs. 3R–T); only at P42 is OPL reduced to 4 μm. In *rd10/Sig1R^−/−^* mice, OPL thickness decreased dramatically as early as P28 to approximately 5 μm (Fig. 3R), suggesting significantly diminished input to neurons within the INL.

### Histologic Assessment Confirms Accelerated PRC Loss in *rd10/Sig1R^−/−^* Retina

WT, *rd10*, and *rd10/Sig1R^−/−^* retinas (P15–P42) were evaluated microscopically (Figs. 4A–G) and subjected to systematic morphometric analysis (Figs. 4H–Y). At P15, there was little difference in TRT of WT versus *rd10* or *rd10/Sig1R^−/−^* mice (Fig. 4A). By P18, however, significant differences emerged, which were detectable morphometrically. *rd10* mouse TRT was reduced compared with WT as early as P18 and thickness decreased gradually with age (Figs. 4H–M). *rd10/Sig1R^−/−^* mice showed pronounced TRT reduction, which differed significantly from *rd10* mice at P21 to P42, confirming SD-OCT results (Fig. 3). ONL-T decreased in *rd10* mice compared with WT by P18 (Fig. 4N), but was more pronounced in *rd10/Sig1R^−/−^* mice beginning at P21 (Fig. 4O). Indeed, from P21 through the oldest age examined (P42) ONL-T in *rd10/Sig1R^−/−^* mice was reduced significantly compared with *rd10* mice (Figs. 4P–S).

**Figure 4:**
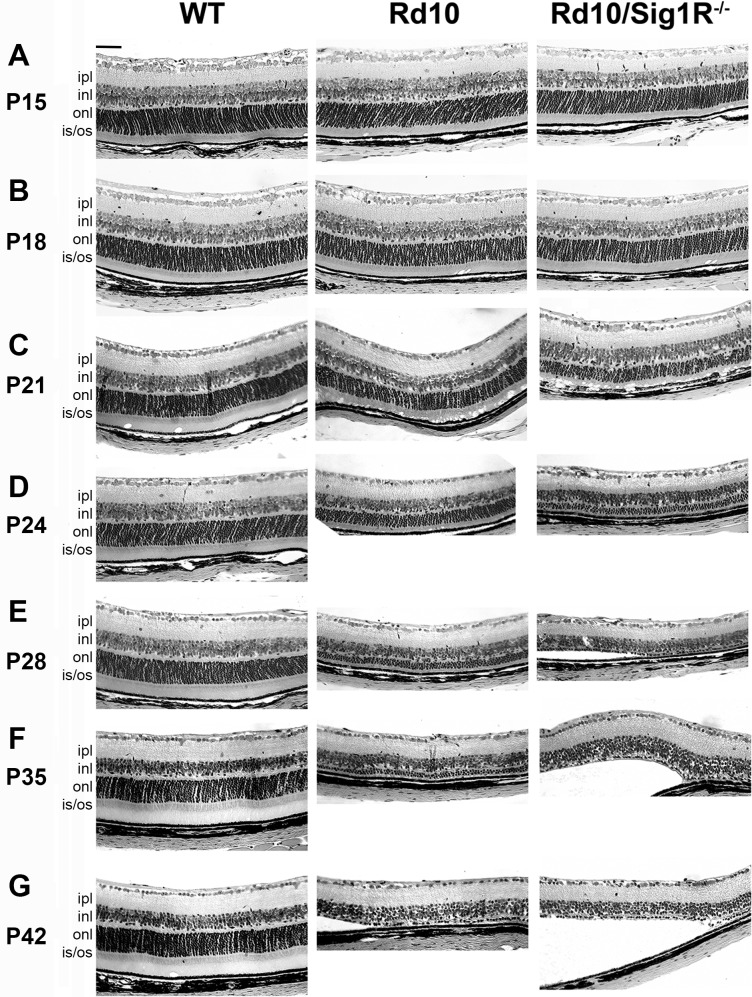
Retinal structure and morphometric analysis. Retinal sections of eyes embedded in JB4 and stained with H&E from WT, rd10, and rd10/Sig1R^−/−^ mice. (A–G) Representative image from WT, rd10, rd10/Sig1R^−/−^ groups at P15 (A), P18 (B), P21 (C), P24 (D), P28 (E), P35 (F) and P42 (G). Note accelerated retinal detachment and paucity of PRC in the ONL in rd10/Sig1R^−/−^ compared with rd10 mice. (H–Y) Morphometric analyses of TRT (H–M), ONL thickness (N–S), and OPL thickness (T–Y) at P18, P21, P24, P28, P35, and P42 separately. gcl, ganglion cell layer; ipl, inner plexiform layer; inl, inner nuclear layer; opl, outer plexiform layer; onl, outer nuclear layer; is, inner segment; os, outer segment; rpe, retinal pigment epithelium. Data are the mean ± SEM of measurements from six to nine mice per group. *P < 0.05; **P < 0.01; ***P < 0.001. Scale bar: 50 μm. Numbers of mice used in the analysis are provided in Supplementary Table S1.

**Figure 4:**
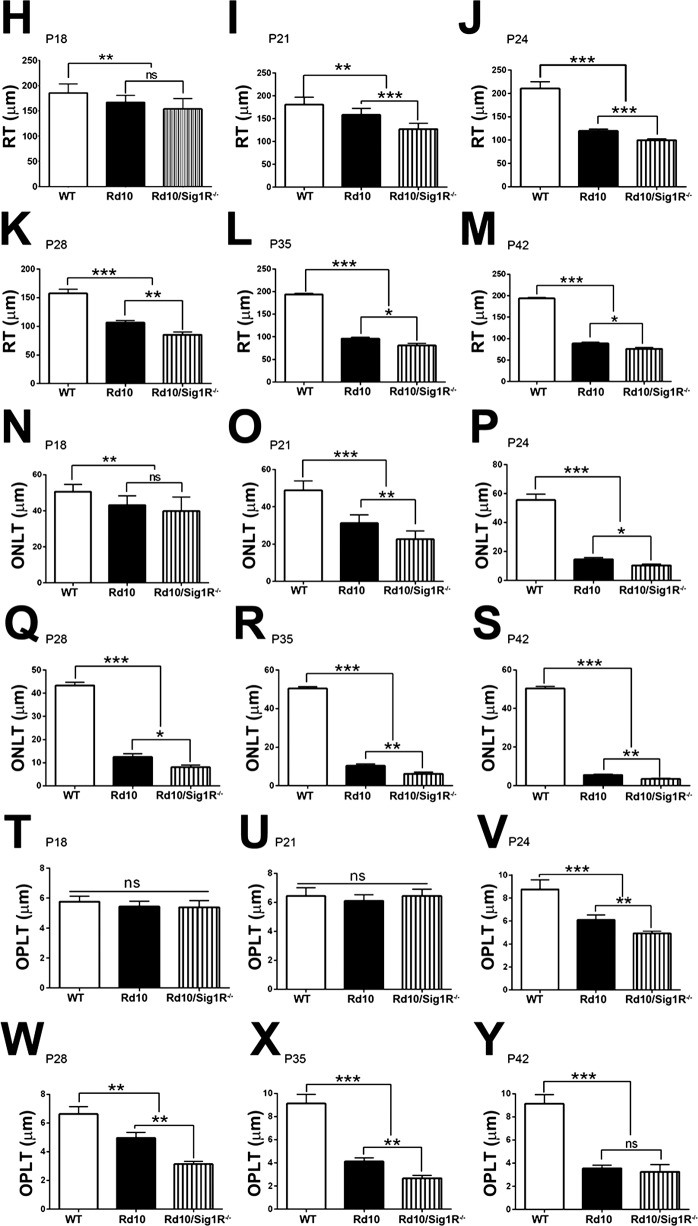
Continued.

WT retinas have approximately 10 to 12 rows of PRC nuclei (Figs. 4A–G). *rd10* mice had 11 to 12 rows through P18, as did *rd10/Sig1R^−/−^* mice (Figs. 4A, 4B). Beginning at P21, there was a rapid decline in rows of nuclei in *rd10* mice: P21, approximately seven to eight rows; P24, approximately five to six rows; P28, approximately three to four rows; P35, three to four rows; P42, one to two rows. The loss of PRC rows was more pronounced in *rd10/Sig1R^−/−^* mice: P21, six to seven rows; P24, three to four rows; P28, approximately one row. Beyond P28, PRC loss was so severe in *rd10/Sig1R^−/−^* retinas that it was not possible to count rows of cells; thus, we counted PRCs per 225-μm retinal length (field of view of ×20 microscope objective). PRC number in P28 *rd10/Sig1R^−/−^* retinas averaged 80.5 ± 10.3, which was approximately 35% less than *rd10* retinas (125 ± 20.2). At P35, PRC number in *rd10/Sig1R^−/−^* retinas averaged 41.3 ± 13.9, which was approximately 60% less than *rd10* retinas (107.1 ± 8.6). Clearly, PRC loss accelerates markedly in *rd10* mice in the absence of Sig1R.

OPL-T was similar between WT, *rd10*, and *rd10/Sig1R^−/−^* mice at P18 and P21 (Figs. 4T, 4U); however, differences in this synaptic layer were detectable by P24; OPL-T was significantly less in *rd10/Sig1R^−/−^* mice compared with *rd10* mice through P35 (Figs. 4V–Y). Despite the accelerated loss of outer retinal layers (ONL, OPL) there was no change in numbers of cells in the ganglion cell layer in *rd10* or *rd10/Sig1R^−/−^* retinas over the period studied (data not shown).

### Sig1R Deletion Accelerates Cone PRC Death in *rd10* Mice

**Figure 5:**
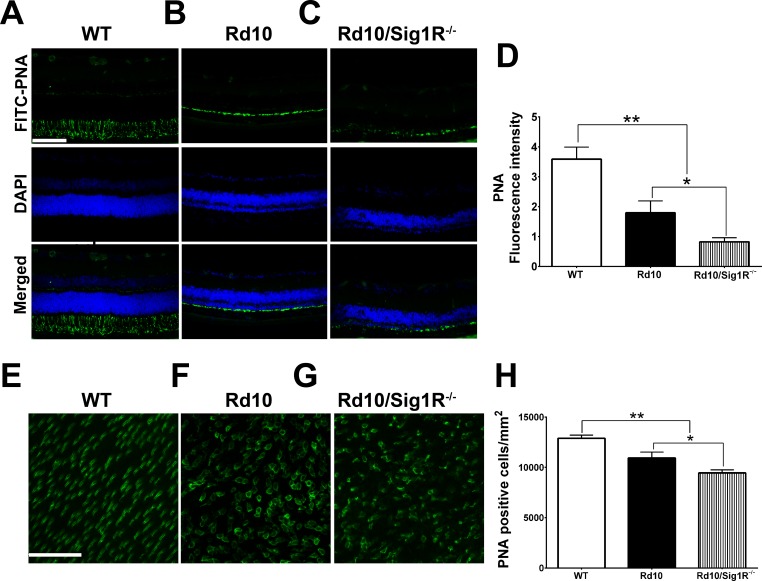
Immunodetection of cone PRCs. (A–C) Retinal cryosections subjected to PNA immunolabeling from retinas of WT (A), rd10 (B), and rd10/Sig1R^−/−^ (C) mice at P35. (D) Quantification of PNA fluorescence. (E–G) Representative PNA-immunolabeled retinal flatmounts from WT (E), rd10 (F), and rd10/Sig1R^−/−^ (G) mice. (H) Quantification of PNA-positive cells. Data are the mean ± SEM of four assays from six to eight mice (cryosections) and from 7 to 10 mice (flatmounts) (Supplementary Table S1). *P < 0.05; **P < 0.01. Scale bars: 100 μm in (A–C); 50 μm in (E–G). Nuclei are labeled with 4′,6-diamidino-2-phenylindole (DAPI) (blue). Details about numbers of mice used in the analysis are provided in Supplementary Table S1, and details about antibodies are provided in Supplementary Table S2.

The genetic mutation in *rd10* mice is specific for rod cGMP-PDE; however, cones are lost subsequently with age.^6–8^ We used FITC-PNA labeling to quantify cones in retinas of WT, *rd10*, and *rd10/Sig1R^−/−^* mice at P35. In WT mice, PNA labeling is robust in inner/outer segments (Fig. 5A); it is diminished in *rd10* mice (Fig. 5B), and it is barely detectable in *rd10/Sig1R^−/−^* mice (Fig. 5C). Quantification of fluorescence intensity confirmed accelerated cone loss in *rd10/Sig1R^−/−^* mice (Fig. 5D). Additionally, retinal flatmounts optically sectioned by laser-scanning confocal microscopy to detect cone PRCs (Figs. 5E–G) showed fewer cones in *rd10/Sig1R^−/−^* mice compared with *rd10* or WT mice (Fig. 5H).

### Sig1R Deletion Accelerates Gliosis in *rd10* Mice

**Figure 6:**
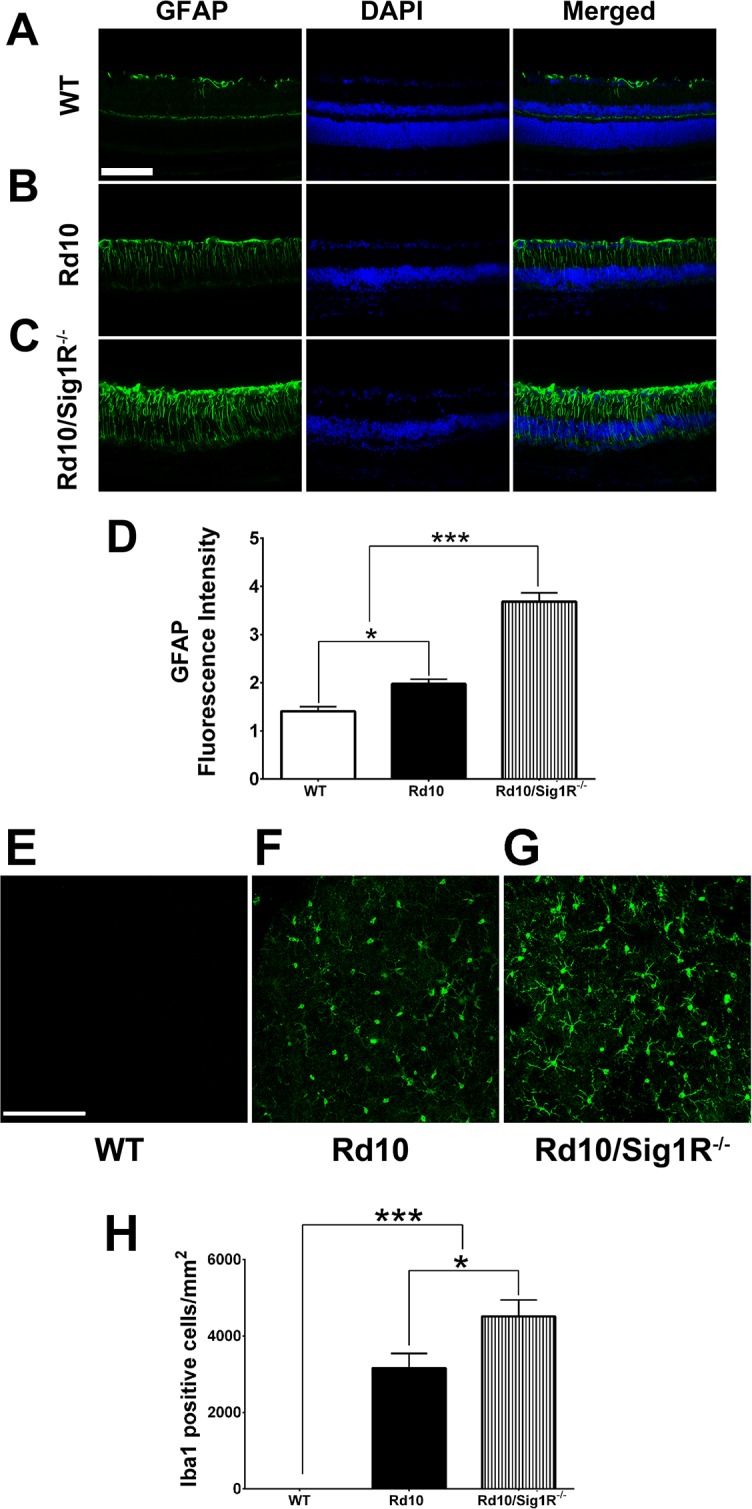
Assessment of glial cell activation. (A–C) Immunodetection of GFAP (green) in retinal cryosections from WT (A), rd10 (B), and rd10/Sig1R^−/−^ mice (C) at P35. Nuclei are labeled with DAPI (blue). (D) Quantification of fluorescence intensity in WT, rd10, and rd10/Sig1R^−/−^ mice. Data are the mean ± SEM (three or four assays) from six to eight mice per group. *P < 0.05; ***P < 0.001 (Supplementary Table S1). (E–G) Retinal flatmounts immunolabeled with Iba-1 from WT (E), rd10 (F), and rd10/Sig1R^−/−^ (G) mice. (H) Quantification of Iba-1 positive cells in retinal flatmounts. Data are the mean ± SEM (three or four assays) from six to eight mice per group. *P < 0.05; ***P < 0.001 (Supplementary Table S1). Calibration bars: (A–C): 100 μm, (E–G): 50 μm. Details about antibodies are provided in Supplementary Table S2.

Increased levels of GFAP, reflecting reactive gliosis in retina, is a sensitive indicator of retinal stress^47^ and has been reported in *rd10* retina.^9,48,49^ In WT, GFAP was restricted to the innermost retina reflecting astrocyte labeling (Fig. 6A). Retinas of *rd10* mice showed distinct radial labeling consistent with activation of Müller glial cells (Fig. 6B). Much more intense radial GFAP labeling was observed in *rd10/Sig1R^−/−^* retinas (Fig. 6C). Quantification of fluorescence intensity confirmed accelerated gliosis in *rd10/Sig1R^−/−^* retinas versus *rd10* (Fig. 6D).

Microglia, resident retinal immune cells, are often reactive in degenerative and inflammatory retinal diseases, including RP.^50^ Microglial activation has been reported in *rd10* mice.^51,52^ When activated, microglia secrete neurotoxic factors, including reactive oxygen and nitrogen species. To detect microglial activation in WT, *rd10*, and *rd10/Sig1R^−/−^* mice, flatmounted retinal preparations were subjected to Iba-1 labeling, which was minimal in WT (Fig. 6E). It increased significantly in *rd10* retinas (Fig. 6F), but was greater still in *rd10/Sig1R^−/−^* retinas (Fig. 6G). The ramified appearance of Iba-1 labeled microglia in *rd10/Sig1R^−/−^* retinas (Fig. 6G) was more intense than observed in *rd10* retinas (Fig. 6F), consistent with a highly activated state. Data are quantified in Figure 6H.

### Sig1R Deletion Increases Oxidative and ER Stress in *rd10* Mice

**Figure 7:**
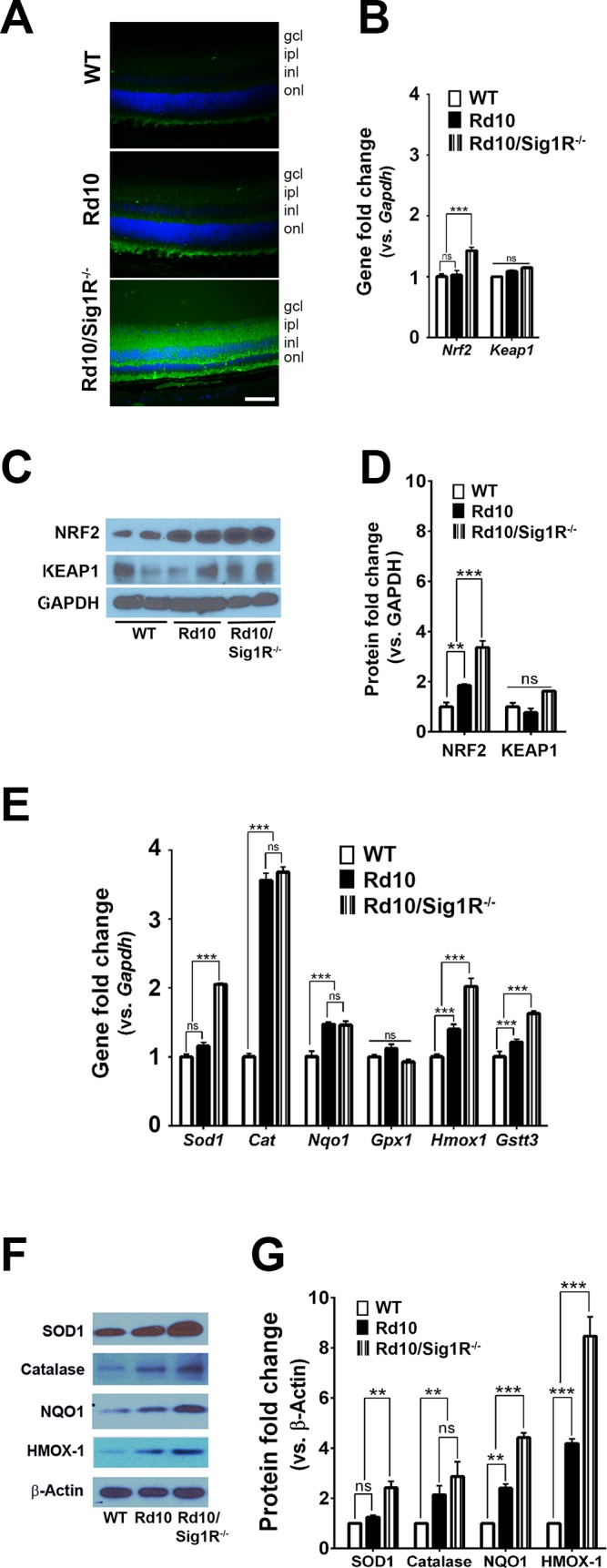
Assessment of oxidative stress–related genes and proteins. Immunodetection of CM-H_2_DCFDA (green fluorescence on reaction with reactive oxygen species) in retinal cryosections of (A) WT, rd10, and rd10/Sig1R^−/−^ at P21. Nuclei are labeled with DAPI (blue). Calibration bar: 50 μm. RNA was isolated from neural retinas and subjected to quantitative RT-PCR (qRT-PCR) analysis of (B) Nrf2, Keap1. Neural retinas harvested from WT, rd10, and rd10/Sig1R^−/−^ mice at P21 were used for isolation of protein. Representative immunoblots detecting (C) NRF2 and KEAP1. (D) Band densities of NRF2 and KEAP1 quantified densitometrically and expressed as fold change versus glyceraldehyde 3-phosphate dehydrogenase (GAPDH). RNA was isolated from neural retinas and subjected to qRT-PCR analysis of (E) Sod1, Catalase, Nqo1, Gpx1, Hmox1, and Gstt3. (F) Neural retinas harvested from WT, rd10, and rd10/Sig1R^−/−^ mice at P21 were used for isolation of protein. Representative immunoblots detecting SOD1, Catalase, NQO1, HMOX1. (G) Band densities of SOD1, Catalase, NQO1, HMOX1 quantified densitometrically and expressed as fold change versus β-Actin. Primer pairs for PCR studies are provided in Supplementary Table S3, and antibodies for immunodetection are provided in Supplementary Table S2. Data are the mean ± SEM of three assays from three different mice retinas in each group. *P < 0.05; **P < 0.01; ***P < 0.001.

A pathogenic feature common in retinal disease is oxidative stress. Rod PRCs are prime oxygen consumers in retina. When they die, as in RP, inner retinal capillaries undergo atrophy. However, choroidal vessels supplying the outer retina with nutrients and oxygen do not autoregulate, thus oxygen from this vascular source is unchecked leading to a hyperoxic retinal environment.^2,53^ Oxidative damage is implicated in PRC death in *rd10* mice.^54,55^ We evaluated oxidative stress in mutant mice at P21 because PRC loss was significantly greater at this age in *rd10/Sig1R^−/−^* versus *rd10* mice (Figs. 1–4). We subjected retinal cryosections to CM-H_2_DCFDA, which fluoresces green in the presence of reactive oxygen species. Minimal fluorescence was detected in WT retinas; a modest level of green fluorescence was noticeable in P21 *rd10* mice, whereas a stronger green fluorescence was observed in *rd10/Sig1R^−/−^* mice (Fig. 7A). A common cellular response to oxidative stress is upregulation of *Nrf2*, which encodes NRF2 (nuclear factor erythroid 2-related factor 2), a molecule that regulates transcription of more than 500 antioxidant/cytoprotective genes.^56,57^ In the absence of overt stress, NRF2 is retained in cytoplasm by KEAP1 (kelch-like ECH-associated protein 1). Under cellular stress, KEAP1 releases NRF2, which translocates to the nucleus to activate “antioxidant response elements” of genes that encode cell defense proteins/enzymes. Under such situations, levels of NRF2 can increase, as has been reported in *rd10* mice by age P42 when PRC loss is considerable.^9^ We examined levels of *Nrf2* and *Keap1* at P21. There was no increase in *Nrf2* or *Keap1* expression in *rd10* mice compared with WT, but there was a significant increase in *Nrf2* in *rd10/Sig1R^−/−^* mice (Fig. 7B). NRF2 protein levels increased significantly in *rd10* retinas, but were greater still in *rd10/Sig1R^−/−^* retinas (Figs. 7C, 7D). Expression of several NRF2-regulated antioxidant genes, including *Sod1, Cat, Nqo1, Hmox1*, and *Gstt3*, was greater in *rd10/Sig1R^−/−^* compared with *rd10* mice (Fig. 7E). Protein levels of SOD1, NQO1, and HMOX-1 were elevated in *rd10/Sig1R^−/−^* versus *rd10* retinas (Figs. 7F, 7G). The data indicate upregulation of the NRF2 antioxidant pathway in mutants lacking Sig1R compared with those expressing Sig1R.

**Figure 8:**
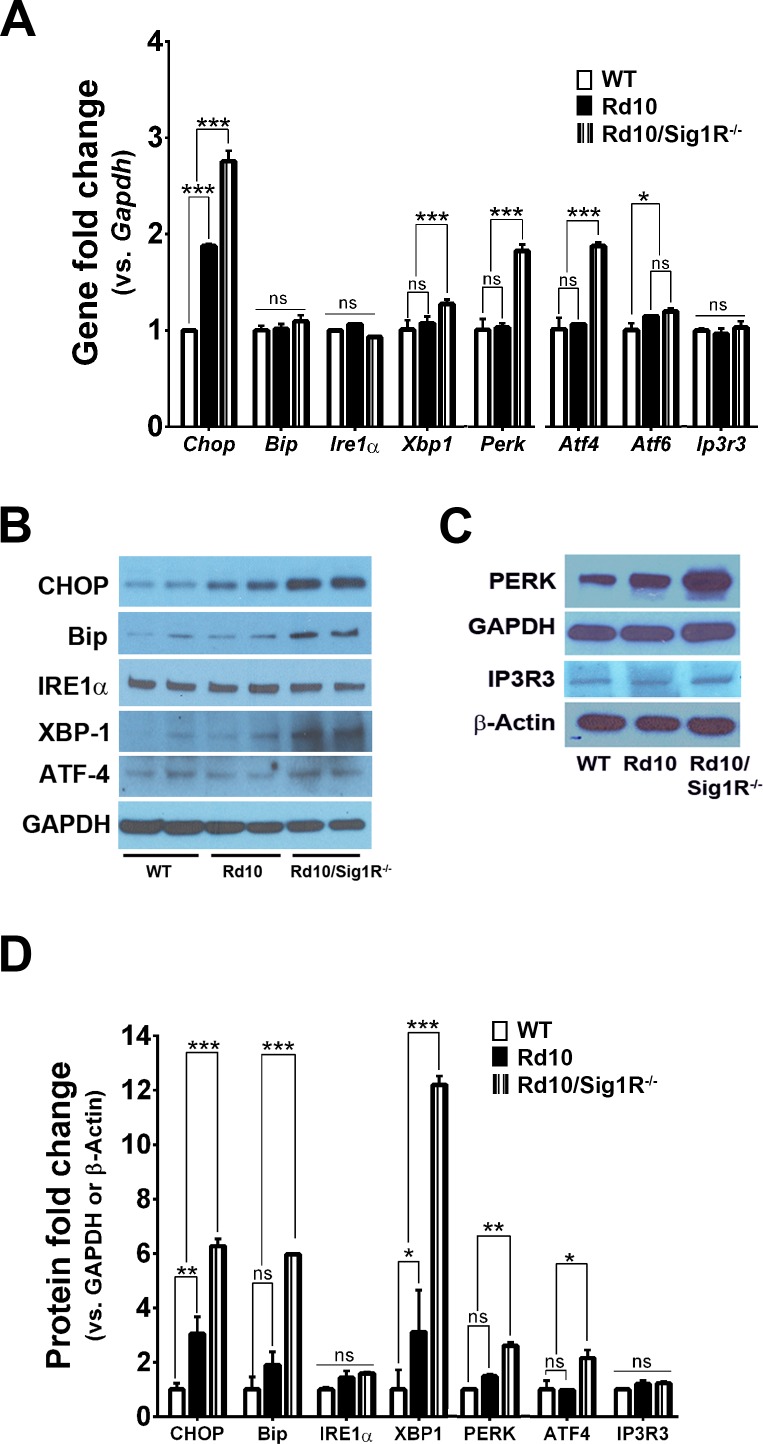
Assessment of ER stress–related genes and proteins. (A) RNA was isolated from neural retinas and subjected to quantitative real-time RT-PCR analysis of Ire1α, Xbp1, Atf4, Chop, Bip, Perk, Ip3r3, and Atf6. Primer pairs are listed in Supplementary Table S3. Data are the mean ± SEM of three assays from three different mice retinas in each group. *P < 0.05; **P < 0.01; ***P < 0.001. (B, C) Neural retinas harvested from WT, rd10, and rd10/Sig1R^−/−^ mice at P21 were used for isolation of protein. Representative immunoblots detecting IRE1α, XBP1, ATF4, CHOP, BiP, PERK, IP3R3, and p-eIF2α/eIF2α are shown in (B) and (C). GAPDH and β-Actin were the internal controls, separately. (D) Band densities, quantified densitometrically and expressed as fold change versus GAPDH or β-Actin. Data are the mean ± SEM of three or four assays from different retina of WT, rd10, and rd10/Sig1R^−/−^ mice. *P < 0.05; **P < 0.01; ***P < 0.001.

Apoptotic death of PRCs is a feature of retinal degenerative diseases, including RP. C/EBP homologous 68 protein (CHOP) is a proapoptic transcription factor activated in some models of RP.^58^ Expression of *Chop* mRNA in P21 *rd10/Sig1R^−/−^* mice was greater than *rd10* mice (Fig. 8A), which was accompanied by increased CHOP protein levels (approximately 5-fold increase in *rd10/Sig1R^−/−^* mice versus WT compared with 2-fold increase in *rd10* mice) (Figs. 8B, 8D).

Increased levels of CHOP and apoptosis of cells are characteristic of the unfolded protein response (UPR) in which misfolded proteins are retained in the endoplasmic reticulum (ER). The initiation of UPR reflects altered levels of binding protein/glucose regulated protein 78 (BiP/GRP78), which in turn releases several ER integral membrane proteins: inositol requiring 1 (IRE1), pancreatic ER eIF2α kinase (PERK), and activating transcription factor 6 (ATF6). We examined BiP, IRE1, PERK, and ATF6 at the mRNA and protein levels in *rd10* and *rd10/Sig1R^−/−^* retinas.

Although there was no change in gene expression of BiP/GRP78, at P21 in *rd10* or *rd10/Sig1R^−/−^* mice compared with WT mice (Fig. 8A), there was a significant increase in BiP/GRP78 protein in *rd10/Sig1R^−/−^* retinas (4-fold greater than WT or *rd10*) (Figs. 8B, 8D). Expression of *Ire1α* was unchanged also (at the gene and protein levels); however, there was a marked increase in *Xbp1* and its encoded protein in *rd10/Sig1R^−/−^* retinas compared with *rd10* or WT (Figs. 8A, 8B, 8D). XBP1, which is downstream of *Ire1α*, is a potent transcriptional activator of many UPR target genes,^59^ thus its increased level in *rd10/Sig1R^−/−^* retinas versus *rd10* is noteworthy. Expression of PERK was elevated significantly at the gene and protein levels in *rd10/Sig1R^−/−^* retinas compared with *rd10* and WT retinas (Figs. 8A, 8C, 8D). Expression of ATF4, a protein downstream of PERK, whose activation can increase levels of chaperones and restore redox homeostasis, was also elevated slightly, albeit significantly, in *rd10/Sig1R^−/−^* retinas compared with *rd10* or WT (Figs. 8A, 8B, 8D). Expression of ATF6 was not changed between *rd10* and *rd10/Sig1R^−/−^* retinas (Fig. 8A). Owing to the putative role of Sig1R in chaperoning inositol phosphate 3 receptor 3 (IP3R3) at the ER/mitochondrion interface ensuring proper Ca^2+^ signaling,^60^ we examined IP3R3 expression; however, we detected no change in mRNA or protein levels in *rd10* or *rd10/Sig1R^−/−^* retinas compared with WT (Figs. 8A, 8C, 8D).

## Discussion

Activation of Sig1R confers protective benefits in several models of neurodegenerative diseases,^21–23^ including retinal diseases.^29–38^ (+)-PTZ, a high-affinity Sig1R ligand, is the prototypical compound used to evaluate Sig1R activation.^61^ Remarkable cone rescue reported in the *rd10* model of retinal degeneration requires the presence of Sig1R for (+)-PTZ to confer neuroprotection because, when absent, (+)-PTZ does not preserve cone function.^9^ Whether Sig1R can modulate rate or severity of *rd10* PRC degeneration had not been addressed. The present work investigated this and provides evidence that *rd10* PRC degeneration, especially cone dysfunction, is accelerated when Sig1R is absent.

Deletion of Sig1R (with no other known genetic mutations) does not have immediate deleterious consequences on retina. Rather, a late-onset RGC loss, with subtler dysfunction, manifests as mice age.^40^ Aside from RGC loss, the retina in *Sig1R^−/−^* mice appears intact with no discernible loss of INL or ONL neurons. If *Sig1R^−/−^* mice are subjected to additional stress, such as diabetes or optic nerve crush, RGC loss accelerates.^41–43^ To address whether Sig1R has a role in maintaining retinal neurons other than RGCs, we took advantage of an early-onset retinal pathology that predominantly affects outer retina. The highly predictable PRC degeneration in *rd10* mice allowed comprehensive assessment of effects of Sig1R deletion on rate/severity of outer retinal degeneration functionally and histologically. The analysis of *rd10*/*Sig1R^−/−^* mice revealed accelerated PRC degeneration and loss of cone function much earlier than *rd10/Sig1R^+/+^* mice. Interestingly, the RGC loss characteristic of *Sig1R^−/−^* mice at approximately 9 to 12 months did not manifest at young ages (P15–P42) examined in *rd10*/*Sig1R^−/−^* mice.

Functional tests used in this study included ERG and OCT. As expected, rod function (measured by scotopic ERG) was reduced markedly in *rd10* mice compared with WT by P28. On the other hand, cone function was robust at this age (approximately 50% of WT). In P28 *rd10/Sig1R^−/−^* mice, however, cone function was reduced to approximately 25% that of WT. The marked reduction in cone function in *rd10/Sig1R^−/−^* mice was confirmed using natural noise stimuli in photopic ERG analysis. Assessment of retina using SD-OCT allowed measurements of retinal layer thicknesses in vivo. ONL-T at P21 in *rd10* mice was approximately 75% of WT, whereas in *rd10/Sig1R^−/−^* mice, it was approximately 25% of WT. This represents a markedly accelerated PRC loss in mutant mice lacking Sig1R. Functional retinal assessments were complemented by histologic and morphometric analyses. We observed accelerated PRC loss in *rd10/Sig1R^−/−^* mice compared with *rd10* mice as early as P21. When we used immunohistochemical methods to specifically label cone PRCs, we found significantly reduced labeling in *rd10/Sig1R^−/−^* mice. We also detected increased gliosis in *rd10/Sig1R^−/−^* mice in the form of increased GFAP labeling of Müller glial cells and increased Iba-1 levels indicative of increased microglial activity compared with *rd10* mice. Taken collectively, the data indicate that in the absence of Sig1R, PRC degeneration and gliosis accelerate in this mutant model.

We acknowledge that the SD-OCT analysis showed separation of the neural retina from RPE at an earlier age (P28) in *rd10/Sig1R^−/−^* mice compared with *rd10* mice. There could be ischemia occurring in *rd10/Sig1R^−/−^* mice, which could severely affect PRC viability. The present study did not investigate retinal vasculature, but this would be an intriguing line of future work. It is noteworthy that the loss of PRCs in the ONL of the *rd10/Sig1R^−/−^* mice was significantly greater than *rd10* mice at P18 and P21, ages that preceded detection of the neural retina-RPE separation. The decrease in inner segment/outer segment thickness is also of note, especially because the inner segment houses mitochondria and the absence of Sig1R may affect these organelles in the PRCs. Investigating this was beyond the scope of the present work, but warrants further study.

Retinal neuron death in degenerative diseases, such as RP, involves a variety of pathologic mechanisms, most notably oxidative stress^2–4^ and ER stress.^58,62^ As a putative molecular chaperone, Sig1R has been studied for its role in modulating these mechanisms; experimental evidence suggests that Sig1R activation attenuates both types of stress.^9,13,36,63^ Oxidative stress leads to activation of the NRF2-KEAP1 pathway.^64^ We observed a modest increase in NRF2 levels in P21 *rd10* mice compared with WT, but significantly higher NRF2 levels in age-matched *rd10/Sig1R^−/−^* mice. This was accompanied by increased levels of antioxidant proteins Sod1, Catalase, NQO1, and Hmox1 in *rd10/Sig1R^−/−^* mice compared with *rd10* mice. The data suggest that if Sig1R is deficient, oxidative stress in an existing retinopathy increases. We find it intriguing and somewhat counterintuitive that NRF2 levels increase with concomitant increase of downstream antioxidants in the *rd10/Sig1R^−/−^* mutant mouse retina, because this pathway is considered a protective cellular mechanism to combat stress levels. The expectation would be that increased production of antioxidants would improve cellular survival. Perhaps when oxidative stress overwhelms the retina, as occurs in *rd10/Sig1R^−/−^* mice, the pathway remains upregulated. It is noteworthy that in an earlier study we also observed increased NRF2 levels and upregulation of antioxidants in *rd10* mouse retinas, but administration of (+)-PTZ (i.e., activation of Sig1R) for many weeks gradually attenuated NRF2 and antioxidant gene expression, suggesting that oxidative stress had been reduced.^9^ The data raise intriguing questions as to whether Sig1R has any role in regulating aspects of the NRF2 antioxidant pathway.

In our study, we also observed an increase in ER stress proteins BiP/GRP78, XBP1, and PERK in *rd10/Sig1R^−/−^* mice compared with *rd10* mice. The most dramatic increase was in the ER-stress–related downstream protein XBP1, which is associated with ER-associated degradation of proteins.^59^ In P21 *rd10* mice, XBP1 was 2-fold greater than WT mice; however, it was 11-fold greater in *rd10/Sig1R^−/−^* mice. XBP1 is regulated by IRE1 and earlier reports demonstrate that Sig1R stabilizes IRE1 at the mitochondrion-associated ER membrane.^13^ A major consequence of excessive ER stress is apoptosis, and CHOP is a key mediator of apoptosis.^65^ As expected, because PRCs are beginning to die at P21, CHOP was elevated at the mRNA and protein levels in *rd10* mice at P21; however, levels in *rd10/Sig1R^−/−^* mice were significantly greater than *rd10* mice at this age. Thus, assessment of oxidative and ER stress reveals increased levels of both in the absence of Sig1R. Future studies could examine additional pathways that are likely involved in Sig1R-mediated cellular survival, including several proteins known to bind this molecular chaperone.^14^

In summary, there is considerable evidence supporting the role of Sig1R in maintaining ganglion cell function within retina.^29,20,32,33,39,66,67^ The present work provides compelling evidence that Sig1R has a role in maintaining PRCs as well particularly in a preexisting PRC-degenerative condition. Retinal phenotype, especially cone survival and function, in an existing model of PRC degeneration worsened markedly in the absence of Sig1R. The findings provide strong in vivo data supporting the role of Sig1R as a modulator of retinal cell survival and may have far-reaching implications for Sig1R as a pathologic mediator in many organ systems.

## Supplementary Material

Supplement 1

Supplement 2

Supplement 3

## References

[1] Dowling JE. Restoring vision to the blind: introduction. *Trans Vis Sci Tech*. 2014; 3 7: 2. 10.1167/tvst.3.7.2 PMC4314996 25653886

[2] Guadagni V, Novelli E, Piano I, Gargini C, Strettoi E. Pharmacological approaches to retinitis pigmentosa: a laboratory perspective. *Prog Retin Eye Res*. 2015; 48: 62– 81. 26113212 10.1016/j.preteyeres.2015.06.005

[3] Komeima K, Rogers BS, Lu L, Campochiaro PA. Antioxidants reduce cone cell death in a model of retinitis pigmentosa. *Proc Natl Acad Sci U S A*. 2006; 103: 11300– 11305. 16849425 10.1073/pnas.0604056103 PMC1544081

[4] Shen J, Yang X, Dong A, Oxidative damage is a potential cause of cone cell death in retinitis pigmentosa. *J Cell Physiol*. 2005; 203: 457– 464. 15744744 10.1002/jcp.20346

[5] Daiger SP, Sullivan LS, Bowne SJ. Genes and mutations causing retinitis pigmentosa. *Clin Genet*. 2013; 84: 132– 141. 23701314 10.1111/cge.12203 PMC3856531

[6] Chang B, Hawes NL, Hurd RE, Davisson MT, Nusinowitz S, Heckenlively JR. Retinal degeneration mutants in the mouse. *Vision Res*. 2002; 42: 517– 525. 11853768 10.1016/s0042-6989(01)00146-8

[7] Chang B, Hawes NL, Pardue MT, Two mouse retinal degenerations caused by missense mutations in the beta-subunit of rod cGMP phosphodiesterase gene. *Vision Res*. 2007; 47: 624– 633. 17267005 10.1016/j.visres.2006.11.020 PMC2562796

[8] Gargini C, Terzibasi E, Mazzoni F, Strettoi E. Retinal organization in the retinal degeneration 10 (rd10) mutant mouse: a morphological and ERG study. *J Comp Neurol*. 2007; 500: 222– 238. 17111372 10.1002/cne.21144 PMC2590657

[9] Wang J, Saul A, Roon P, Smith SB. Activation of the molecular chaperone, sigma 1 receptor, preserves cone function in a murine model of inherited retinal degeneration. *Proc Natl Acad Sci U S A*. 2016; 113: E3764– E3772. 27298364 10.1073/pnas.1521749113 PMC4932934

[10] Schmidt HR, Zheng S, Gurpinar E, Koehl A, Manglik A, Kruse AC. Crystal structure of the human σ1 receptor. *Nature*. 2016; 532: 527– 530. 27042935 10.1038/nature17391 PMC5550834

[11] Hayashi T, Su TP. Sigma-1 receptor chaperones at the ER-mitochondrion interface regulate Ca(2+) signaling and cell survival. *Cell*. 2007; 131: 596– 610. 17981125 10.1016/j.cell.2007.08.036

[12] Ruscher K, Wieloch T. The involvement of the sigma-1 receptor in neurodegeneration and neurorestoration. *J Pharmacol Sci*. 2015; 127: 30– 35. 25704015 10.1016/j.jphs.2014.11.011

[13] Mori T, Hayashi T, Hayashi E, Su TP. Sigma-1 receptor chaperone at the ER-mitochondrion interface mediates the mitochondrion-ER-nucleus signaling for cellular survival. *PLoS One*. 2013; 8: e76941. 24204710 10.1371/journal.pone.0076941 PMC3799859

[14] Su TP, Su TC, Nakamura Y, Tsai SY. The Sigma-1 receptor as a pluripotent modulator in living systems. *Trends Pharmacol Sci*. 2016; 37: 262– 278. 26869505 10.1016/j.tips.2016.01.003 PMC4811735

[15] Al-Saif A, Al-Mohanna F, Bohlega S. A mutation in sigma-1 receptor causes juvenile amyotrophic lateral sclerosis. *Ann Neurol*. 2011; 70: 913– 919. 21842496 10.1002/ana.22534

[16] Prause J, Goswami A, Katona I, Altered localization, abnormal modification and loss of function of Sigma receptor-1 in amyotrophic lateral sclerosis. *Hum Mol Genet*. 2013; 22: 1581– 1600. 23314020 10.1093/hmg/ddt008

[17] Fehér Á, Juhász A, László A, Association between a variant of the sigma-1 receptor gene and Alzheimer's disease. *Neurosci Lett*. 2012; 517: 136– 139. 22561649 10.1016/j.neulet.2012.04.046

[18] Huang Y, Zheng L, Halliday G, Genetic polymorphisms in sigma-1 receptor and apolipoprotein E interact to influence the severity of Alzheimer's disease. *Curr Alzheimer Res*. 2011; 8: 765– 770. 21605063 10.2174/156720511797633232

[19] Mishina M, Ishiwata K, Ishii K, Function of sigma1 receptors in Parkinson's disease. *Acta Neurol Scand*. 2005; 112: 103– 107. 16008536 10.1111/j.1600-0404.2005.00432.x

[20] Mavlyutov TA, Epstein ML, Verbny YI, Lack of sigma-1 receptor exacerbates ALS progression in mice. *Neuroscience*. 2013; 240: 129– 134. 23458708 10.1016/j.neuroscience.2013.02.035 PMC3665351

[21] Peviani M, Salvaneschi E, Bontempi L, Neuroprotective effects of the Sigma-1 receptor (S1R) agonist PRE-084, in a mouse model of motor neuron disease not linked to SOD1 mutation. *Neurobiol Dis*. 2014; 62: 218– 232. 24141020 10.1016/j.nbd.2013.10.010

[22] Hedskog L, Pinho CM, Filadi R, Modulation of the endoplasmic reticulum-mitochondria interface in Alzheimer's disease and related models. *Proc Natl Acad Sci U S A*. 2013; 110: 7916– 7921. 23620518 10.1073/pnas.1300677110 PMC3651455

[23] Francardo V, Bez F, Wieloch T, Nissbrandt H, Ruscher K, Cenci MA. Pharmacological stimulation of sigma-1 receptors has neurorestorative effects in experimental parkinsonism. *Brain*. 2014; 137: 1998– 2014. 24755275 10.1093/brain/awu107

[24] Jiang G, Mysona B, Dun Y, Expression, subcellular localization, and regulation of sigma receptor in retinal Müller cells. *Invest Ophthalmol Vis Sci*. 2006; 47: 5576– 5582. 17122151 10.1167/iovs.06-0608 PMC3724475

[25] Mavlyutov TA, Epstein M, Guo LW. Subcellular localization of the sigma-1 receptor in retinal neurons—an electron microscopy study. *Sci Rep*. 2015; 5: 10689. 26033680 10.1038/srep10689 PMC4649997

[26] Ola MS, Moore P, El-Sherbeny A, Expression pattern of sigma receptor 1 mRNA and protein in mammalian retina. *Brain Res Mol Brain Res*. 2001; 95: 86– 95. 11687279 10.1016/s0169-328x(01)00249-2 PMC3742362

[27] Mavlyutov TA, Nickells RW, Guo LW. Accelerated retinal ganglion cell death in mice deficient in the Sigma-1 receptor. *Mol Vis*. 2011; 17: 1034– 1043. 21541278 PMC3084245

[28] Ola MS, Moore P, Maddox D, Analysis of sigma receptor (sigmaR1) expression in retinal ganglion cells cultured under hyperglycemic conditions and in diabetic mice. *Brain Res Mol Brain Res*. 2002; 107: 97– 107. 12425939 10.1016/s0169-328x(02)00444-8 PMC3773709

[29] Smith SB, Duplantier J, Dun Y, In vivo protection against retinal neurodegeneration by sigma receptor 1 ligand (+)-pentazocine. *Invest Ophthalmol Vis Sci*. 2008; 49: 4154– 4161. 18469181 10.1167/iovs.08-1824 PMC2562718

[30] Zhao L, Chen G, Li J, An intraocular drug delivery system using targeted nanocarriers attenuates retinal ganglion cell degeneration. *J Control Release*. 2017; 247: 153– 166. 28063892 10.1016/j.jconrel.2016.12.038 PMC5323250

[31] Liu LL, Deng QQ, Weng SJ, Yang XL, Zhong YM. Activation of the sigma receptor 1 modulates AMPA receptor-mediated light-evoked excitatory postsynaptic currents in rat retinal ganglion cells. *Neuroscience*. 2016; 332: 53– 60. 27373906 10.1016/j.neuroscience.2016.06.045

[32] Zhao J, Mysona BA, Qureshi A, (+)-Pentazocine reduces NMDA-induced murine retinal ganglion cell death through a σR1-dependent mechanism. *Invest Ophthalmol Vis Sci*. 2016; 57: 453– 461. 26868747 10.1167/iovs.15-18565 PMC4758298

[33] Mueller BH II, Park Y, Ma HY, Sigma-1 receptor stimulation protects retinal ganglion cells from ischemia-like insult through the activation of extracellular-signal-regulated kinases 1/2. *Exp Eye Res*. 2014; 128: 156– 169. 25305575 10.1016/j.exer.2014.10.007

[34] Campana G, Bucolo C, Murari G, Spampinato S. Ocular hypotensive action of topical flunarizine in the rabbit: role of sigma 1 recognition sites. *J Pharmacol Exp Ther*. 2002; 303: 1086– 1094. 12438531 10.1124/jpet.102.040584

[35] Vogler S, Winters H, Pannicke T, Wiedemann P, Reichenbach A, Bringmann A. Sigma-1 receptor activation inhibits osmotic swelling of rat retinal glial (Müller) cells by transactivation of glutamatergic and purinergic receptors. *Neurosci Lett*. 2016; 610: 13– 18. 26499958 10.1016/j.neulet.2015.10.042

[36] Wang J, Shanmugam A, Markand S, Zorrilla E, Ganapathy V, Smith SB. Sigma 1 receptor regulates the oxidative stress response in primary retinal Müller glial cells via NRF2 signaling and system xc(−), the Na(+)-independent glutamate-cystine exchanger. *Free Radic Biol Med*. 2015; 86: 25– 36. 25920363 10.1016/j.freeradbiomed.2015.04.009 PMC4554890

[37] Zhao J, Ha Y, Liou GI, Gonsalvez GB, Smith SB, Bollinger KE. Sigma receptor ligand, (+)-pentazocine, suppresses inflammatory responses of retinal microglia. *Invest Ophthalmol Vis Sci*. 2014; 55: 3375– 3384. 24812552 10.1167/iovs.13-12823 PMC4042630

[38] Shimazawa M, Sugitani S, Inoue Y, Tsuruma K, Hara H. Effect of a sigma-1 receptor agonist, cutamesine dihydrochloride (SA4503), on photoreceptor cell death against light-induced damage. *Exp Eye Res*. 2015; 132: 64– 72. 25616094 10.1016/j.exer.2015.01.017

[39] Saul AB, Still AE. Multifocal electroretinography in the presence of temporal and spatial correlations and eye movements. *Vision*. 2016; 1: 3. 10.3390/vision1010003 PMC6849053 31740628

[40] Ha Y, Saul A, Tawfik A, Late-onset inner retinal dysfunction in mice lacking sigma receptor 1 (σR1). *Invest Ophthalmol Vis Sci*. 2011; 52: 7749– 7760. 21862648 10.1167/iovs.11-8169 PMC3183986

[41] Ha Y, Saul A, Tawfik A, Zorrilla EP, Ganapathy V, Smith SB. Diabetes accelerates retinal ganglion cell dysfunction in mice lacking sigma receptor 1. *Mol Vis*. 2012; 18: 2860– 2870. 23233788 PMC3519370

[42] Wang J, Cui X, Roon P, Smith SB. Role of sigma 1 receptor in retinal degeneration of the Ins2Akita/+ murine model of diabetic retinopathy. *Invest Ophthalmol Vis Sci*. 2016; 57: 2770– 2781. 27206247 10.1167/iovs.15-18995 PMC4884059

[43] Mavlyutov TA, Nickells RW, Guo LW. Accelerated retinal ganglion cell death in mice deficient in the Sigma-1 receptor. *Mol Vis*. 2011; 17: 1034– 1043. 21541278 PMC3084245

[44] Sabino V, Cottone P, Parylak SL, Steardo L, Zorrilla EP. Sigma-1 receptor knockout mice display a depressive-like phenotype. *Behav Brain Res*. 2009; 198: 472– 476. 19100292 10.1016/j.bbr.2008.11.036 PMC2667953

[45] Chang B, Hurd R, Wang J, Nishina P. Survey of common eye diseases in laboratory mouse strains. *Invest Ophthalmol Vis Sci*. 2013; 54: 4974– 4981. 23800770 10.1167/iovs.13-12289 PMC3723375

[46] Hasegawa T, Ikeda HO, Nakano N, Changes in morphology and visual function over time in mouse models of retinal degeneration: an SD-OCT, histology, and electroretinography study. *Jpn J Ophthalmol*. 2016; 60: 111– 125. 26729343 10.1007/s10384-015-0422-0

[47] Reichenbach A, Bringmann A. New functions of Müller cells. *Glia*. 2013; 61: 651– 678. 23440929 10.1002/glia.22477

[48] Ly A, Merl-Pham J, Priller M, Proteomic profiling suggests central role of STAT signaling during retinal degeneration in the rd10 mouse model. *J Proteome Res*. 2016; 15: 1350– 1359. 26939627 10.1021/acs.jproteome.6b00111

[49] Martínez-Fernández de la Cámara C, Hernández-Pinto AM, Olivares-González L, Adalimumab reduces photoreceptor cell death in a mouse model of retinal degeneration. *Sci Rep*. 2015; 5: 11764. 26170250 10.1038/srep11764 PMC4501000

[50] Karlstetter M, Scholz R, Rutar M, Wong WT, Provis JM, Langmann T. Retinal microglia: just bystander or target for therapy? *Prog Retin Eye Res*. 2015; 45: 30– 57. 25476242 10.1016/j.preteyeres.2014.11.004

[51] Arroba AI, Alvarez-Lindo N, van Rooijen N, de la Rosa EJ. Microglia-mediated IGF-I neuroprotection in the rd10 mouse model of retinitis pigmentosa. *Invest Ophthalmol Vis Sci*. 2011; 52: 9124– 9130. 22039242 10.1167/iovs.11-7736

[52] Zabel MK, Zhao L, Zhang Y, Microglial phagocytosis and activation underlying photoreceptor degeneration is regulated by CX3CL1-CX3CR1 signaling in a mouse model of retinitis pigmentosa. *Glia*. 2016; 64: 1479– 1491. 27314452 10.1002/glia.23016 PMC4958518

[53] Yu DY, Cringle SJ. Retinal degeneration and local oxygen metabolism. *Exp Eye Res*. 2005; 80: 745– 751. 15939030 10.1016/j.exer.2005.01.018

[54] Usui S, Oveson BC, Iwase T, Overexpression of SOD in retina: need for increase in H2O2-detoxifying enzyme in same cellular compartment. *Free Radic Biol Med*. 2011; 51: 1347– 1354. 21736939 10.1016/j.freeradbiomed.2011.06.010 PMC3163708

[55] Obolensky A, Berenshtein E, Lederman M, Zinc-desferrioxamine attenuates retinal degeneration in the rd10 mouse model of retinitis pigmentosa. *Free Radic Biol Med*. 2011; 51: 1482– 1491. 21824515 10.1016/j.freeradbiomed.2011.07.014

[56] Sporn MB, Liby KT. NRF2 and cancer: the good, the bad and the importance of context. *Nat Rev Cancer*. 2012; 12: 564– 571. 22810811 10.1038/nrc3278 PMC3836441

[57] Gorrini C, Harris IS, Mak TW. Modulation of oxidative stress as an anticancer strategy. *Nat Rev Drug Discov*. 2013; 12: 931– 947. 24287781 10.1038/nrd4002

[58] Lin JH, Li H, Yasumura D, IRE1 signaling affects cell fate during the unfolded protein response. *Science*. 2007; 318: 944– 949. 17991856 10.1126/science.1146361 PMC3670588

[59] Glimcher LH. XBP1: the last two decades. *Ann Rheum Dis*. 2010; 69 Suppl 1: i67– i71. 19995749 10.1136/ard.2009.119388

[60] Watanabe S, Ilieva H, Tamada H, Mitochondria-associated membrane collapse is a common pathomechanism in SIGMAR1- and SOD1-linked ALS. *EMBO Mol Med*. 2016; 8: 1421– 1437. 27821430 10.15252/emmm.201606403 PMC5167132

[61] de Costa BR, Bowen WD, Hellewell SB, Synthesis and evaluation of optically pure [3H]-(+)-pentazocine, a highly potent and selective radioligand for sigma receptors. *FEBS Lett*. 1989; 251: 53– 58. 2568952 10.1016/0014-5793(89)81427-9

[62] Gorbatyuk MS, Knox T, LaVail MM, Restoration of visual function in P23H rhodopsin transgenic rats by gene delivery of BiP/Grp78. *Proc Natl Acad Sci U S A*. 2010; 107: 5961– 5966. 20231467 10.1073/pnas.0911991107 PMC2851865

[63] Nguyen L, Lucke-Wold BP, Mookerjee SA, Role of sigma-1 receptors in neurodegenerative diseases. *J Pharmacol Sci*. 2015; 127: 17– 29. 25704014 10.1016/j.jphs.2014.12.005

[64] Johnson DA, Johnson JA. Nrf2—a therapeutic target for the treatment of neurodegenerative diseases. *Free Radic Biol Med*. 2015; 88: 253– 267. 26281945 10.1016/j.freeradbiomed.2015.07.147 PMC4809057

[65] Iurlaro R, Muñoz-Pinedo C. Cell death induced by endoplasmic reticulum stress. *FEBS J*. 2016; 283: 2640– 2652. 26587781 10.1111/febs.13598

[66] Mueller BH, Park Y, Daudt DR, Sigma-1 receptor stimulation attenuates calcium influx through activated L-type voltage gated calcium channels in purified retinal ganglion cells. *Exp Eye Res*. 2013; 107: 21– 31. 23183135 10.1016/j.exer.2012.11.002

[67] Tchedre KT, Huang RQ, Dibas A, Krishnamoorthy RR, Dillon GH, Yorio T. Sigma-1 receptor regulation of voltage-gated calcium channels involves a direct interaction. *Invest Ophthalmol Vis Sci*. 2008; 49: 4993– 5002. 18641291 10.1167/iovs.08-1867

