# Supplementary material for: Absence of Sigma 1 Receptor Accelerates Photoreceptor Cell Death in a Murine Model of Retinitis Pigmentosa
Source: Invest Ophthalmol Vis Sci. 2017 Sep;58(11):4545–58. doi: 10.1167/iovs.17-21947 (PMC5586962; doi:10.1167/iovs.17-21947)
Supplement: Supplement 1 [file iovs-58-10-56_s01.pdf]

Supplementary Table S1. Numbers and ages of animals used in the study.

Mouse group	n	Age (post-natal days)
ERG analysis of retinal function		
Wild-type	5	28
Rd10	6	28
Rd10/Sig1R$^{-/-}$	8	28
OCT and JB4 morphometric analysis of retinal structure		
Wild-type	4	15
Rd10	4	15
Rd10/Sig1R$^{-/-}$	4	15
Wild-type	4	18
Rd10	4	18
Rd10/Sig1R$^{-/-}$	4	18
Wild-type	8	21
Rd10	8	21
Rd10/Sig1R$^{-/-}$	9	21
Wild-type	4	24
Rd10	4	24
Rd10/Sig1R$^{-/-}$	4	24
Wild-type	8	28
Rd10	8	28
Rd10/Sig1R$^{-/-}$	8	28
Wild-type	8	35
Rd10	9	35
Rd10/Sig1R$^{-/-}$	12	35
Wild-type	8	42
Rd10	8	42
Rd10/Sig1R$^{-/-}$	13	42
Immunohistochemistry and H&E cryosection		
Wild-type	5	28
Rd10	5	28
Rd10/Sig1R$^{-/-}$	5	28
Wild-type	5	35
Rd10	6	35
Rd10/Sig1R$^{-/-}$	6	35
Assessment of microglia activation and PNA-labeled cones in flat mount preparation		
Wild-type	7	35
Rd10	8	35
Rd10/Sig1R$^{-/-}$	10	35
Detection of ER stress and oxidative stress related genes and proteins		
Wild-type	15	21
Rd10	18	21
Rd10/Sig1R$^{-/-}$	20	21
