# Supplementary material for: Absence of Sigma 1 Receptor Accelerates Photoreceptor Cell Death in a Murine Model of Retinitis Pigmentosa
Source: Invest Ophthalmol Vis Sci. 2017 Sep;58(11):4545–58. doi: 10.1167/iovs.17-21947 (PMC5586962; doi:10.1167/iovs.17-21947)
Supplement: Supplement 2 [file iovs-58-10-56_s02.pdf]

Supplementary Table S2. Antibodies used in immunohistochemical, flatmount and immunoblotting studies.

Antibody	Supplier	Dilution
**Primary**		
FITC-conjugated peanut agglutinin (L-7381)	Sigma, St. Louis, MO	1:100
Rabbit anti-GFAP (Z0334)	Dako , Carpentaria, CA	1:500
Rabbit anti-Iba-1 (019-19741)	Wako, VA	1:200
Rabbit anti-IRE1α (14C10)	Cell Signaling, MA	1:1000
Rabbbit anti-XBP1 (12782S)	Cell Signaling, MA	1:1000
Rabbit anti-ATF4 (11815S)	Cell Signaling, MA	1:1000
Mouse anti-CHOP (2895P)	Cell Signaling, MA	1:1000
Rabbit anti-Bip (3177P)	Cell Signaling, MA	1:1000
Rabbit anti-PERK (3192S)	Cell Signaling, MA	1:1000
Mouse anti-GAPDH (MAB374)	EMD Millipore, MA	1:5000
Mouse anti-IP3R3 (610312)	BD Bioscience, CA	1:1000
Rabbit anti-p-eIF2α (3398P)	Cell Signaling, MA	1:1000
Rabbit anti-eIF2α (5324P)	Cell Signaling, MA	1:1000
Rabbit anti-Nrf2 (ab31163)	Abcam, MA	1:800
Goat anti-KEAP1 (SC-15246)	Santa Cruz, CA	1:800
Rabbit anti-SOD1 (SC-11407)	Santa Cruz, CA	1:1000
Rabbit anti-Catalase (SC-50508)	Santa Cruz, CA	1:800
Rabbit anti-NQO1 (ab34173)	Abcam, MA	1:1000
Rabbit anti-HMOX1 (SC-10789)	Santa Cruz, CA	1:500
Rabbit anti-β-Actin (8457P)	Cell Signaling, MA	1:1000
**Secondary**		
Alexa Fluo 488 anti-rabbit IgG (H+L)	Invitrogen Molecular Probes, NY	1:1000
Goat anti-Rabbit IgG-HRP (SC-2004)	Santa Cruz, CA	1:1000
Goat anti-Mouse IgG-HRP (SC-2005)	Santa Cruz, CA	1:1000
Donkey anti-Goat IgG-HRP (SC-2020)	Santa Cruz, CA	1:1000
