# Supplementary material for: Absence of Sigma 1 Receptor Accelerates Photoreceptor Cell Death in a Murine Model of Retinitis Pigmentosa
Source: Invest Ophthalmol Vis Sci. 2017 Sep;58(11):4545–58. doi: 10.1167/iovs.17-21947 (PMC5586962; doi:10.1167/iovs.17-21947)
Supplement: Supplement 3 [file iovs-58-10-56_s03.pdf]

Supplementary Table S3. Sequences of primers used for real-time Q-RT-PCR

Gene	NCBI Acession Number		Primer Sequence	size (bp)
Ire1a	NM_023913	Forward	*5'- ACACCGACCACCGTATCTCA-3'*	
		Reverse	*5'- CTCAGGATAATGGTAGCCATGTC-3'*	110
Xbp1	NM_013842	Forward	*5'- AGCAGCAAGTGGTGGATTTG-3'*	
		Reverse	*5'-GAGTTTTCTCCCGTAAAAGCTGA-3'*	75
Atf4	NM_009716	Forward	*5'-AAGGAGGAAGACACTCCCTCT-3'*	
		Reverse	*5'-CAGGTGGGTCATAAGGTTTGG-3'*	169
Chop	NM_007837	Forward	*5'-CTGGAAGCCTGGTATGAGGAT-3'*	
		Reverse	*5'-CAGGGTCAAGAGTAGTGAAGGT-3'*	121
Bip	NM_022310	Forward	*5'-ACTTGGGGACCACCTATTCCT-3'*	
		Reverse	*5'-ATCGCCAATCAGACGCTCC-3'*	134
Perk	*NM_010121*	Forward	*5'-AGTCCCTGCTCGAATCTTCCT-3'*	
		Reverse	*5'-TCCCAAGGCAGAACAGATATACC-3'*	125
Ip3r3	*NM_080553*	Forward	*5'-GGGCGCAGAACAACGAGAT-3'*	
		Reverse	*5'-GAAGTTTTGCAGGTCACGGTT-3'*	104
Atf6	NM_001081304	Forward	*5'-AGCGCCCAAGACTCAAACC-3'*	
		Reverse	*5'-CTGTATGCTGATAATCGACTGCT-3'*	109
Nrf2	NM_010902	Forward	*5'-TAGATGACCATGAGTCGCTTGC-3'*	
		Reverse	*5'-GCCAAACTTGCTCCATGTCC-3'*	153
Keap1	NM_016679	Forward	*5'-TGCCCCTGTGGTCAAAGTG-3'*	
		Reverse	*5'-GGTTCGGTTACCGTCCTGC-3'*	104
Sod1	NM_011434	Forward	*5'-AACCAGTTGTGTTGTCAGGAC-3'*	
		Reverse	*5'-CCACCATGTTTCTTAGAGTGAGG-3'*	139
Catalase	NM_009804	Forward	*5'-AGCGACCAGATGAAGCAGTG-3'*	
		Reverse	*5'-TCCGCTCTCTGTCAAAGTGTG-3'*	181
Nqo1	NM_008706	Forward	*5'-AGGATGGGAGGTACTCGAATC-3'*	
		Reverse	*5'-AGGCGTCCTTCCTTATATGCTA-3'*	144
Gpx1	NM_008160	Forward	*5'-AGTCCACCGTGTATGCCTTCT-3'*	
		Reverse	*5'-GAGACGCGACATTCTCAATGA-3'*	105
Hmox1	NM_010442	Forward	*5'-AAGCCGAGAATGCTGAGTTCA-3'*	
		Reverse	*5'-GCCGTGTAGATATGGTACAAGGA-3'*	100
Gstt3	NM_133994	Forward	*5'-GGATGGGGACTTCGTCTTGG-3'*	
		Reverse	*5'-TCAGGAGGTACGGGCTGTC-3'*	219
Gapdh	NM_008084	Forward	*5'-AGGTCGGTGTGAACGGATTTG-3'*	
		Reverse	*5'-TGTAGACCATGTAGTTGAGGTCA-3'*	123
